# Supplementary material for: Chiral Liquid Crystal Microdroplets for Sensing Phospholipid Amphiphiles
Source: Biosensors (Basel). 2022 May 9;12(5):313. doi: 10.3390/bios12050313 (PMC9139120; doi:10.3390/bios12050313)
Supplement: Supplementary file 1 [file biosensors-12-00313-s001.zip › biosensors-1708380-supplementary.pdf]

biosensors

MDPI

Article

Chiral Liquid Crystal Microdroplets for Sensing Phospholipid Amphiphiles

Sepideh Norouzi [1], Jose A. Martinez Gonzalez [2] and Monirosadat Sadati [1,*]

[1] Department of Chemical Engineering, University of South Carolina, Columbia, SC 29208, USA; snorouzi@email.sc.edu
[2] Facultad de Ciencias, Universidad Autónoma de San Luis Potosí, Av. Parque Chapultepec 1570, San Luis Potosí 78210 SLP, Mexico; jose.adrian.martinez@uaslp.mx
* Correspondence: sadati@cec.sc.edu

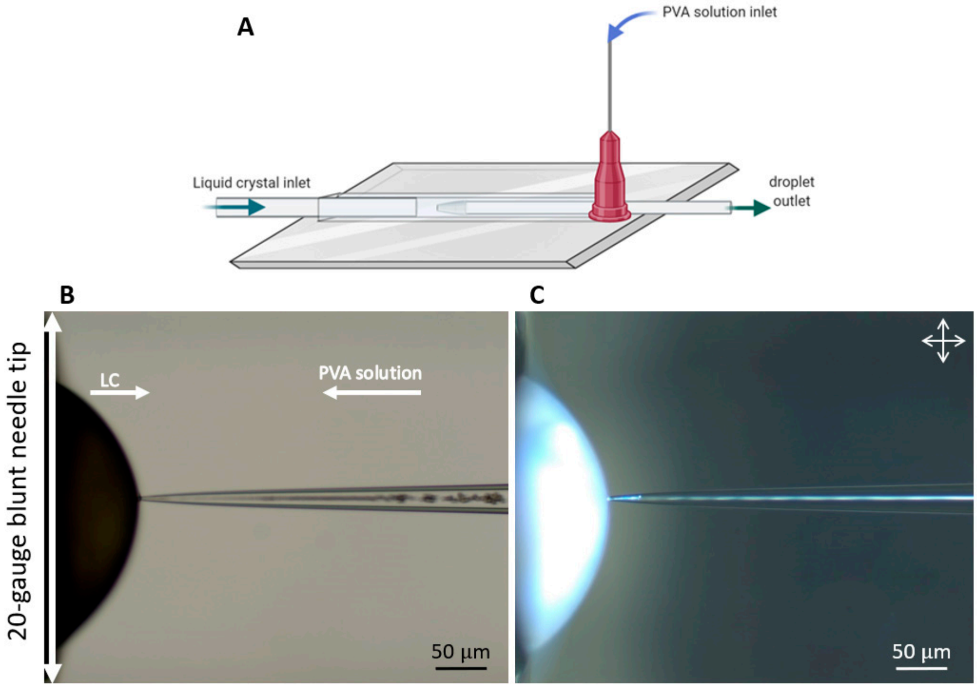

Figure S1. microfluidic device (A) schematic of microfluidic device, (B, C) bright field and POM image in reflection mode of CLC flow inside microfluidic device and droplet production upon LC stream breakup.

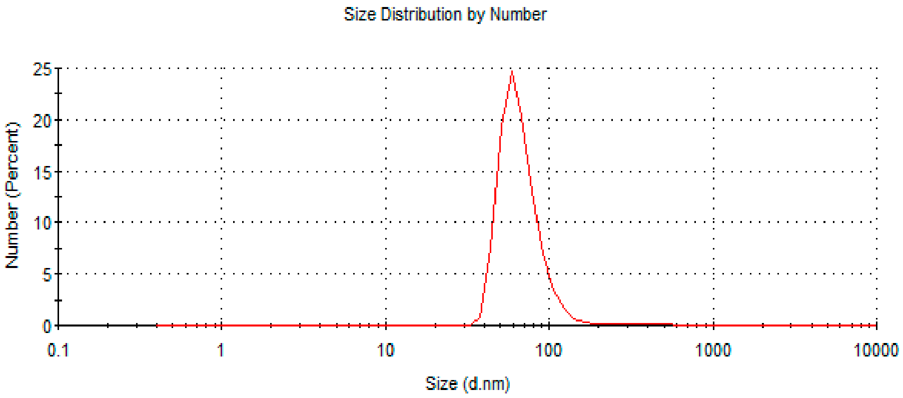

Figure S2. size distribution of 0.5 mM DLPC dispersion.

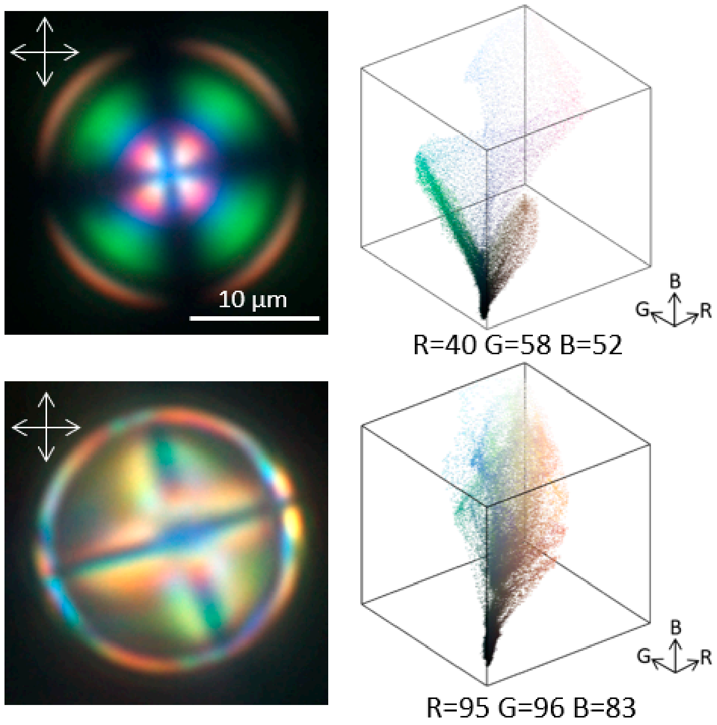

Figure S3. Reflection mode POM images and the colorimetric changes of 20 µm high chirality droplets upon contact with 1 mM DLPC.

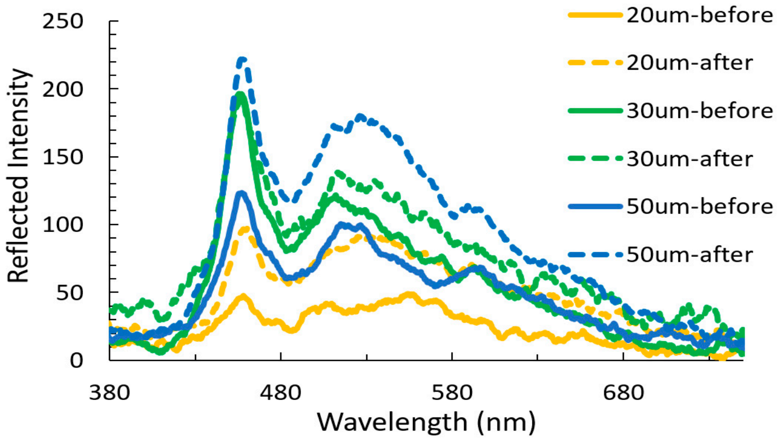

Figure S4. diffused reflection spectra of high chirality droplet before and after DLPC adsorption. spectroscopy readout of high chirality droplets as function of droplet size at phospholipid concentration of 50 µM.

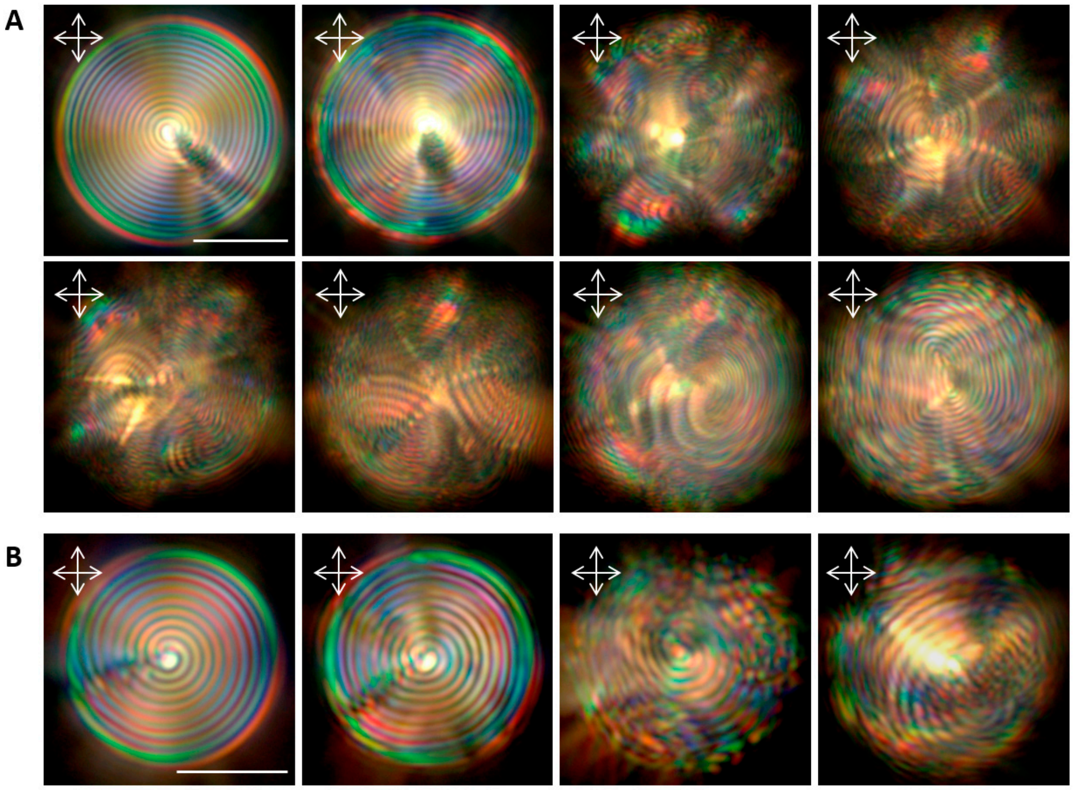

Figure S5. Transmission mode POM images of low chirality droplets upon orientational reconfiguration in contact with DLPC amphiphiles. (A) 50 μm low chirality droplet, (B) 30 μm low chirality droplet.

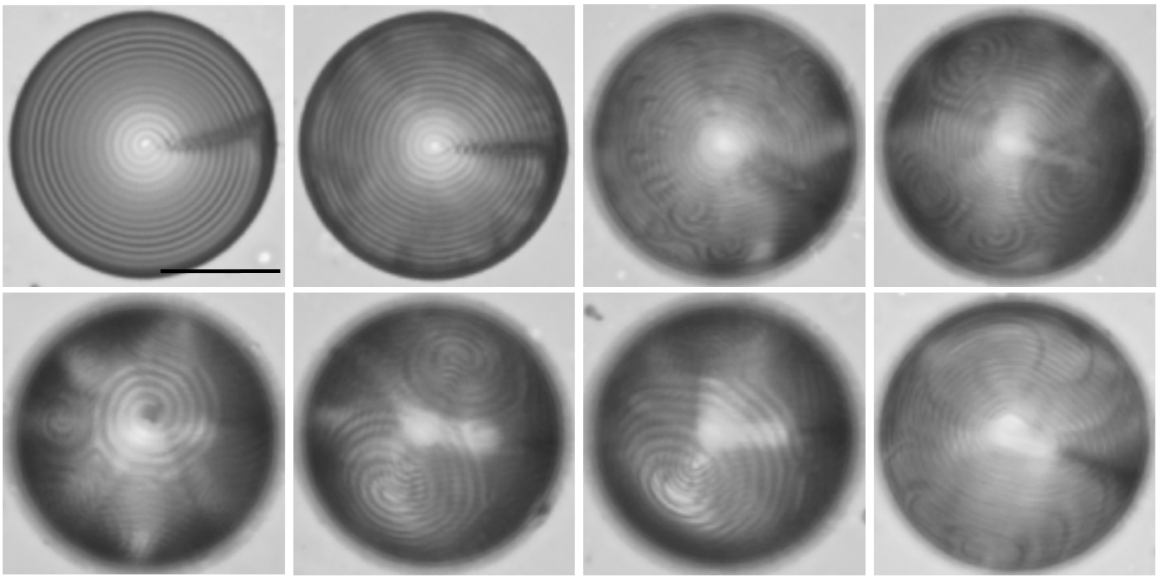

Figure S6. Bright field images of the pitch length change during orientational reconfiguration in 50 μm low chirality droplets.

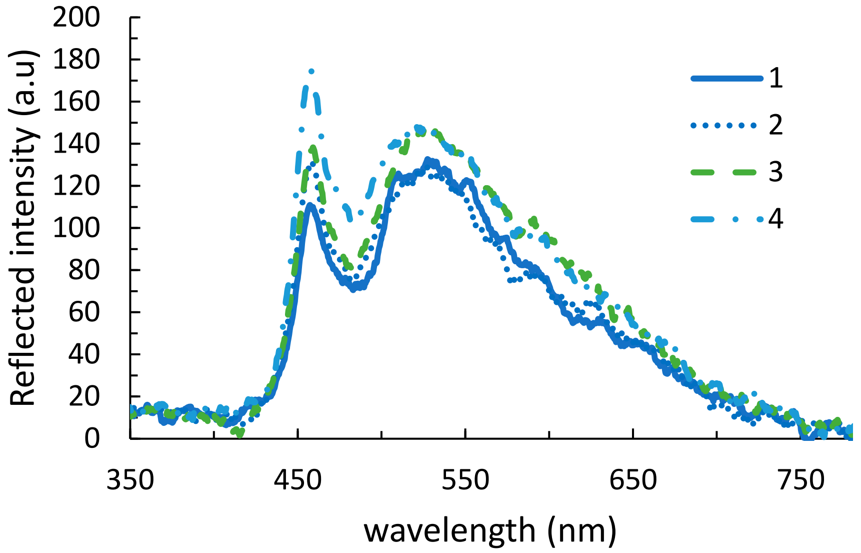

Figure S7. Spectroscopy results of 50 µm low chiral droplets during molecular reorientation in contact with 1mM DLPC.
